# Preliminary Results of Implantation in Animal Model and Osteoblast Culture Evaluation of Prototypes of Biomimetic Multispiked Connecting Scaffold for Noncemented Stemless Resurfacing Hip Arthroplasty Endoprostheses

**DOI:** 10.1155/2013/689089

**Published:** 2013-07-29

**Authors:** Ryszard Uklejewski, Piotr Rogala, Mariusz Winiecki, Andrzej Kędzia, Piotr Ruszkowski

**Affiliations:** ^1^Department of Medical Bioengineering Fundamentals, Institute of Technology, Casimir the Great University, Karola Chodkiewicza 30, 85-064 Bydgoszcz, Poland; ^2^Department of Process Engineering, Institute of Technology and Chemical Engineering, Poznan University of Technology, Marii Sklodowskiej-Curie 2, 60-965 Poznan, Poland; ^3^Department of Spine Surgery, Oncologic Orthopaedics and Traumatology, Poznan University of Medical Sciences, 28 Czerwca 1956 135/147, 61-545 Poznan, Poland; ^4^Department of Clinical Auxology, Poznan University of Medical Sciences, Szpitalna 27/33, 60-572 Poznan, Poland; ^5^Growth Hormone & Growing Skeleton Research Group, Poznan University of Medical Sciences, 28 Czerwca 1956 135/147, 61-545 Poznan, Poland; ^6^Department of Pharmacology, Poznan University of Medical Sciences, Rokietnicka 5A, 60-806 Poznan, Poland

## Abstract

We present the new fixation method for RHA *(resurfacing hip arthroplasty)* endoprostheses by means of the biomimetic multispiked connecting scaffold (MSC-Scaffold). Such connecting scaffold can generate new type of RHA endoprostheses, that is stemless and fixed entirely without cement. The preprototypes of this MSC-Scaffold were manufactured with modern additive laser additive technology (SLM). The pilot surgical implantations in animal model (two laboratory swine) of MSC-Scaffold preprototypes have showed after two months neither implant loosening, migration, and nor other early complications. From the results of performed histopathological evaluation of the periscaffold spikes bone tissue and 10-day culture of human osteoblasts (NHOst) we can conclude that (1) the scaffolding effect was obtained and (2) to improve the osseointegration of the scaffold spikes, their material surface should be physicochemically modified (e.g., with hydroxyapatite). Some histopathological findings in the periscaffold domain near the MSC-Scaffold spikes bases (fibrous connective tissue and metallic particles near the MSC-Scaffold spikes bases edges) prompt considering the necessity to optimize the design of the MSC-Scaffold in the regions of its interspike space near the spikes bases edges, to provide more room for new bone formation in this region and for indispensable post-processing (glass pearl blasting) after the SLM manufacturing.

## 1. Introduction

It is estimated that about 1.3 million endoprostheses are implanted in the world yearly [1]. The main indication for joint replacement is the degenerative disease of articular cartilage (osteoarthrosis, osteoarthritis (OA)). OA affects more than 20% of people aged over 55 years; 1/3 residents of the USA present the clinical symptoms of OA from at least one joint [2]. Osteoarthritis has been placed by WHO in the second place among causes of disability and is an important social problem in many countries. The treatment of choice is the ill-joint replacement with endoprosthesis, that is, the arthroplasty. Because of the degenerated articular cartilage, removed during the hip arthroplasty is not only this damaged cartilage but also, some (often large) part the healthy periarticular trabecular bone of the head and the neck of the femur. Removed cartilage and bone tissue are replaced by a metal artificial joint construction—see the commonly used long-stem endoprostheses of hip or other joints. When transferring mechanical loads, due to the significant differences (10–100x higher) in values of the elastic parameters of endoprostheses metal alloys compared with those of cancellous bone, the bone surrounding the endoprostheses practically does not transfer in the periarticular bone region mechanical loads (*stress shielding* phenomenon)—it results in nonphysiological load transmission and consequently in atrophy and extensive destruction of surrounding periprosthetic bone, loosening and migration of elements of endoprostheses, and even bone fractures [3].

The resurfacing hip arthroplasty (RHA) with the use of the stemless RHA endoprostheses is the epiphyseal trabecular bone preserving alternative to the commonly used long-stem total hip arthroplasty (THA). RHA restores the normal joint biomechanics and close-to-natural load transfer, through the head and the neck of the femur and then along the femoral shaft. Thus the overall stability of the hip joint is improved as compared to the traditional THA and, moreover, the stemless RHA femoral component application saves the proximal femur for an eventual later revision THA with the use of a short-stem or a traditional long-stem endoprosthesis. 

**Figure 1:**
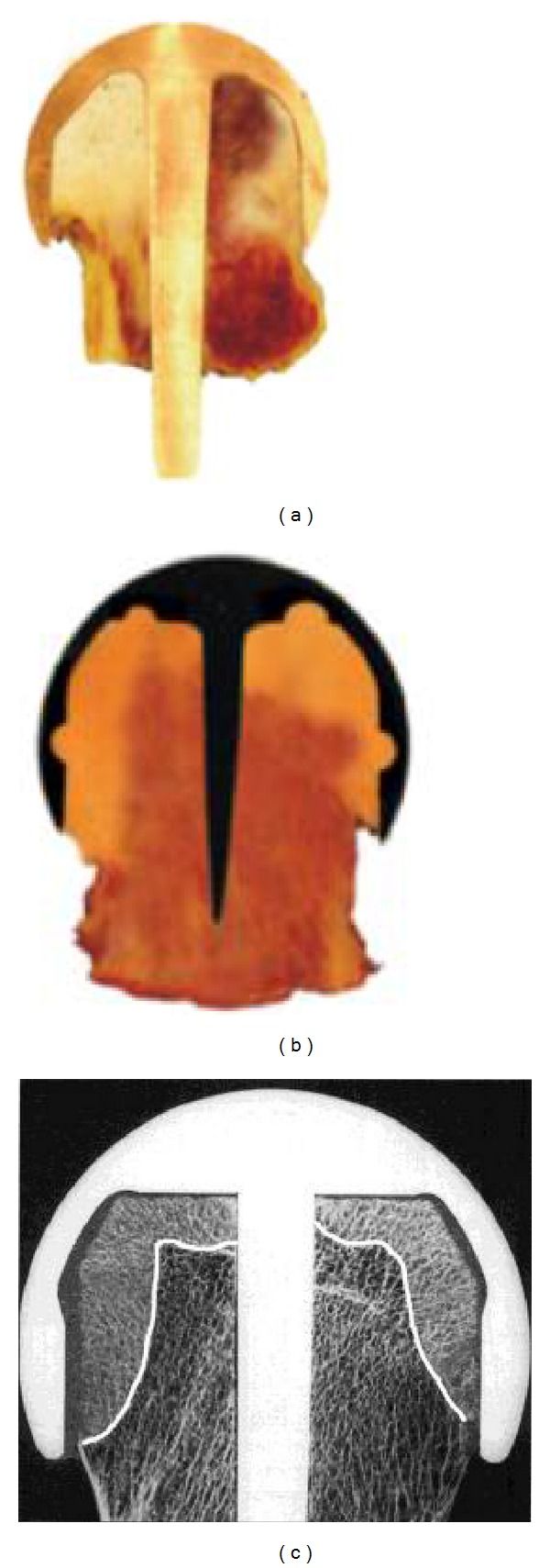
(a), (b) Exemplary cross-sections of three femoral heads with currently used RHAs, where the cups were fixed on femoral heads with the polymethacrylate cement (courtesy of Dr. S. Łazowski, Histopathomorphology Lab., Poznan, Poland); (c) cement pressing the trabecular marrow cavities occupies large volume (bright areas) of the femoral heads, sections showing cement penetration zone in femoral head occupying more than 1/3 of its volume in [4].

**Figure 2:**
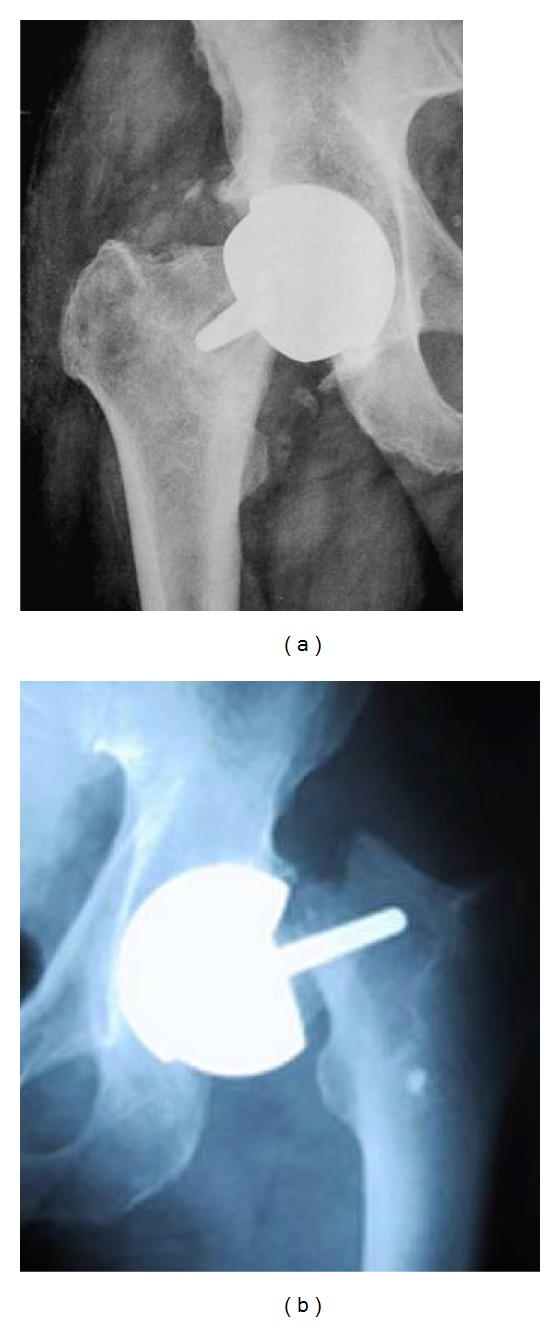
(a) Loosening of femoral component of cemented short-stem endoprostheses [20] and (b) periprosthetic fractures [9] are the main complications of current RHA.

The current worldwide accepted standard fixation method for modern metal-on-metal RHA is a hybrid technique using a cemented short-stem femoral component in combination with an uncemented acetabular component (e.g., Birmingham Hip Implant, Wright Conserve Plus Implant, Cormet 2000 Implant, Zimmer DUROM Implant, ICON Implant, Biomet ReCap Implant, DePuy ASR Implant, and ESKA Resurfacing Implants). Applied cements never guarantee proper and long-lasting RHA endoprostheses fixation. On the one side, the use of cement provides sufficient primary fixation of RHA endoprosthesis femoral component, but on the other one the massive cement penetration into the proximal femoral epiphysis (it often occupies more than 1/3 of its volume, Figure 1, c.f. with [4]) causes regional blood supply insufficiency [5], which may lead to the progressive weakening of the internal bone microstructure and results in failures. Bone resorption, loosening (Figure 2(a)) at bone-cement-implant interface, and migration of the femoral component as well as the periprosthetic fractures (Figure 2(b)) were observed as postoperative complications in numerous clinical studies dealing with the current generation of RHA endoprostheses [6–20]. The periprosthetic necrosis frequently following the cemented short-stem RHA can be actually evidenced by use of the Positron Emission Tomography (PET) [21]. According to de Steiger et al. [22], after excluding infection, the major reasons for revision of primary RHAs are fractures of the femoral neck (43%), loosening/lysis (32%), metal sensitivity (7%), and pain (6%). The most common types of revision are a femoral-only revision (62%), acetabular and femoral revision (29%), and acetabular-only revision (9%).

**Figure 3:**
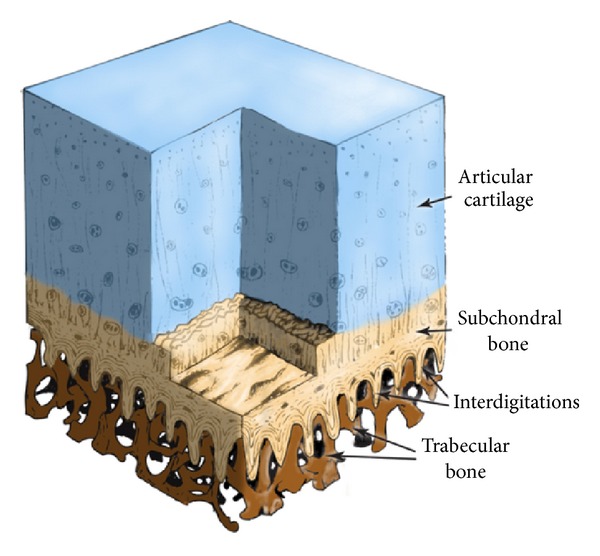
The 3D diagram of articular hyaline cartilage and subchondral bone with interdigitations interlocking with trabeculae of cancellous bone, own scheme of the articular-periarticular biostructures, on the basis of the results of Milz and Putz [34].

*The concept of the method of cementless implantation for RHA endoprostheses* invented by Rogala [23–25] and elaborated in our research team [26–29] is briefly outlined below. The alternative for traditional cement fixation of femoral components of RHA endoprostheses is the entirely cementless fixation by the use of the biomimetic multispiked connecting scaffold (MSC-Scaffold). The MSC-Scaffold prototype was designed by us so that the spikes mimic the interdigitations of subchondral bone, which interpenetrate with the trabeculae of the periarticular cancellous bone and anchor the articular cartilage through the subchondral bone in the periarticular cancellous bone (Figure 3). The biomimetic MSC-Scaffold can be applied in resurfacing arthroplasty of most articular joints (hip, elbow, knee, shoulder, ankle, hand, and foot joints), as well as in implantations of intervertebral discs, because everywhere there the periarticular trabecular bone appears and behaves similarly.

**Figure 4:**
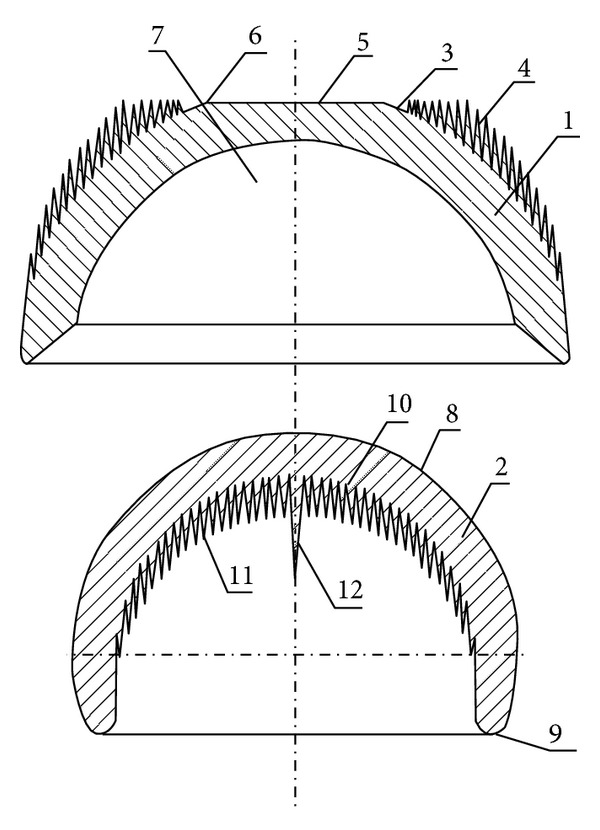
Schematic drawing of acetabulum and head of the entirely cementless total RHA endoprosthesis in cross-section: (1) acetabulum, (2) head, (3) acetabulum spherical boundary surface, (4) acetabulum spikes, (5) circular surface, (6) edge lying in the plane perpendicular to acetabulum axis, (7) pan, (8) external head surface, (9) annular bearing surface, (10) spherical boundary surface, (11) head spikes, and (12) central spike.

The new concept of entirely cementless RHA endoprosthesis includes an acetabulum and a head (Figure 4), while the bearing surfaces are located on round surfaces which include projecting spikes forming MSC-Scaffold. The edges of the bases of adjacent spikes contact each other, and their axes are perpendicular to the surface in which the bearing edge of the acetabulum and the bearing surface of the head lie. Peaks of the projecting spikes of the acetabular cap do not extend beyond the circular plane boundary surface determined by the edge lying on the plane perpendicular to the acetabular axis; however, the head has a bearing surface in annular form with an outer diameter smaller than a diameter of round bowl, which constitutes a spherical cap of the external surface of the head. The length of the acetabulum spikes measured from the base on the boundary surface determines a theoretical spherical surface, concentric to the boundary surface, which crosses the peaks of the spikes. The endoprosthesis acetabulum ((1) in Figure 4) possesses a pan (7) to place the endoprosthesis head (2), which constitutes a part of spherical cap of the external head surface (8). The head has annular bearing surface lying below the transverse axis of the head. On the head spherical boundary surface there are spikes arranged around the central spike with parallel axes to each other, whereas a central spike is coincident with the axis of the head.

**Figure 5:**
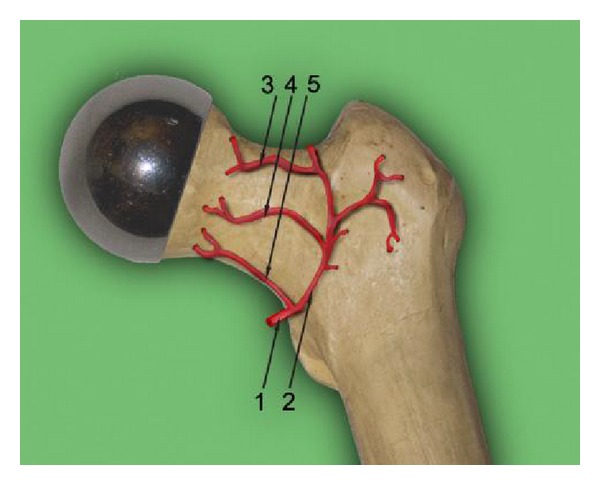
The femoral head component of our prototype of innovating THRA endoprosthesis—designed to preserve the subcapsular arteriae retinacular: superior (3), anterior (4), and inferior (5); (1) a. circumflexa femoris lateralis, (2) ramus ascendens of (1); author's scheme based on the blood supply diagram from [4].

The macrodimensions of the annular bearing part (9) of femoral head component are designed to preserve the posterolateral and medial epiphyseal femoral arteries (subcapsular aa. retinacular) for femoral head; see Figure 5. Consequently, the physiological blood supply and the optimal remodeling potential of the trabecular bone of femoral head are preserved. The filling up of the interspike pore space of MSC-Scaffold by an ingrowing newly formed bone tissue will allow the effective biological fixation in periarticular trabecular bone of the femoral component of the proposed RHA endoprosthesis.

The bioengineering design of the RHA endoprosthesis prototype with the MSC-Scaffold and its generation in the selective laser melting (SLM) technology are presented in our previous papers [27–29]. Such fixation as well as untraditional manufacturing technology does not occur in any of the currently used models of RHA endoprostheses. 

The fixation procedure of RHA endoprosthesis with the biomimetic MSC-Scaffold will proceed in two steps: (1) the mechanical insertion of the endoprosthesis components into the periarticular trabecular bone on the desired osteoconductive level by the operating surgeon and (2) the adaptive bone tissue ingrowth into the interspiked space of the biomimetic MSC-Scaffold. During the penetration into trabecular bone marrow lacunae the spikes of the biomimetic MSC-Scaffold cause the controlled destruction of cancellous bone trabeculae at the desired osteoinductive level, allowing the effective promotion of bone tissue ingrowth into the remaining free space between the spikes (*scaffolding effect*). The filling up of the interspike pore space of the MSC-Scaffold by ingrowing new formed bone tissue will allow bone to remodel to close-to-natural microstructure and shape, what is impossible in the case of currently used cemented short-stem RHA endoprostheses, not to mention the traditional long-stem endoprostheses. After new bone formation, the boundary surface of the acetabulum, and the boundary surface of the head, the circular surface, head annular bearing surface, and the surfaces of the spikes become the bearing surfaces of the endoprosthesis. 

## 2. Materials and Methods

### 2.1. Preprototype of the Biomimetic MSC-Scaffold Characterization

**Figure 6:**
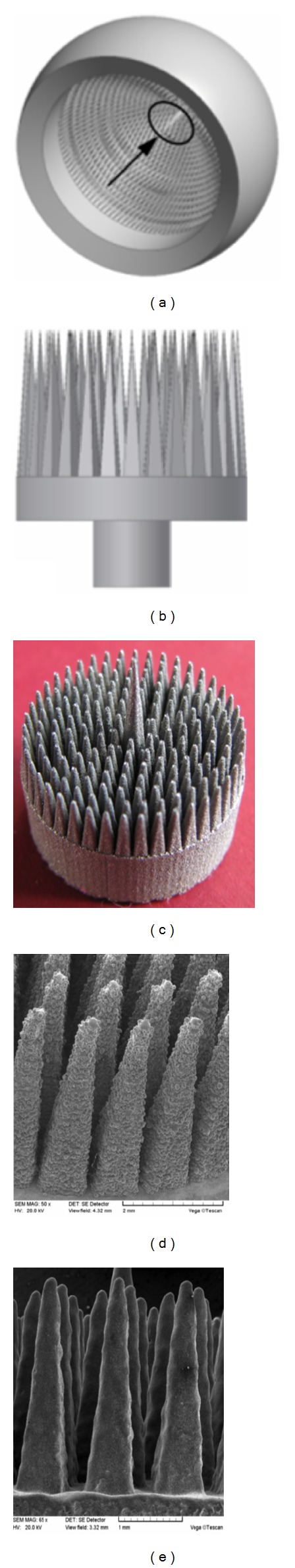
(a) 3D CAD model of the femoral head component of the entirely cementless RHA endoprosthesis with the biomimetic MSC-Scaffold preprototype, (b) biomimetic MSC-Scaffold preprototype representing the fragment indicated with the arrow and ellipse on femoral component, (c) the exemplary SLM manufactured preprototype of the biomimetic MSC-Scaffold, and (d) SEM photograph of the preprototype before and (e) after pearl glass blasting treatment.

In presented research the preprototypes of biomimetic MSC-Scaffold represent the fragment of the femoral component of RHA endoprosthesis located around central spike (Figure 6(a)). The CAD models of the preprototypes were designed in the Autodesk Inventor Professional 9 CAD software in size variant for swine (breed: Polish large white). The preprototypes presented in this paper were comprehensively designed for preliminary preclinical *in vivo* tests on animals and for biological evaluation with human osteoblasts culture presented below, as well as, for biomechanical push-in tests provided to evaluate the implant push-in force and the destruction in bone around implant presented in [30]. In Figure 6(a) the 3D CAD model of RHA endoprosthesis femoral element with biomimetic MSC-Scaffold preprototype is presented, while in Figure 6(b) an exemplary CAD model of the preprototype of the biomimetic MSC-Scaffold, preprototype representing the fragment (indicated with arrow and ellipse) of the femoral component of RHA endoprosthesis femoral component, is shown. The two variants of MSC-Scaffold were designed for implantation in animal model and osteoblast culture evaluation varying with the distance between the spikes bases: 100 *μ*m and 200 *μ*m, both circumferentially and radially and with the external diameter of the MSC-Scaffold preprototype base: *ϕ*10 mm and *ϕ*15 mm, respectively. 

Our preprototypes of biomimetic MSC-Scaffold for RHA endoprostheses were manufactured in the selective laser melting (SLM) technology at the REALIZER II 250 SLM machine (MTT Technologies Group, Germany), and the manufacturing was subcontracted to the SLM Tech Center in Paderborn, Germany, and to the Centre of New Materials and Technologies at West Pomeranian University of Technology, Szczecin, Poland. All preprototypes were manufactured at once of Ti6Al4V powder. The grain size distribution of the powder was from 5 to 50 *μ*m, the mean alloy grain size was 30 *μ*m, and the powder was recommended and provided by the SLM Realizer machine manufacturer. In Figure 6(c) the exemplary preprototype of the biomimetic MSC-Scaffold is presented, and SEM photographs of MSC-Scaffold spikes before and after the pearl glass blasting treatment are showed in Figures 6(d) and 6(e), respectively.

### 2.2. Implantation in Animal Model of Preprototypes of MSC-Scaffold for RHA Endoprostheses

**Figure 7:**
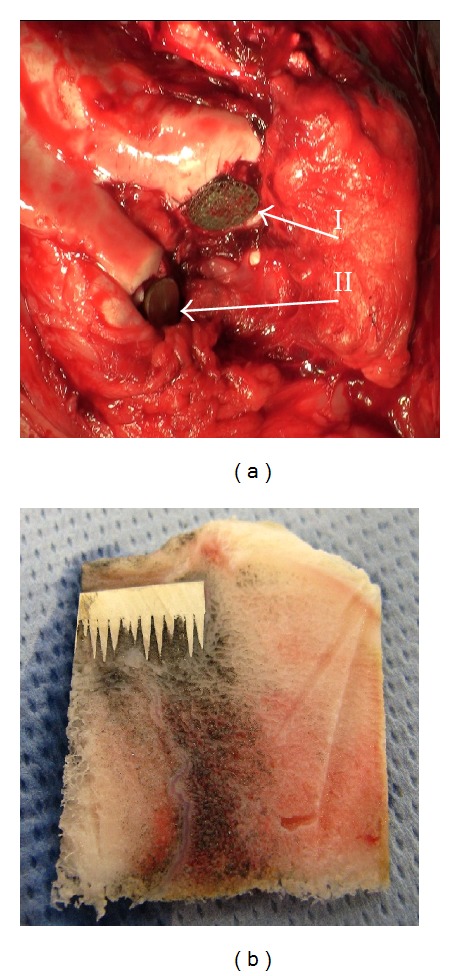
(a) Two variants of MSC-Scaffold preprototypes (I, II) are implanted under the articular cartilage surface of both femoral condyles; (b) the exemplary bone implant 1,5 mm thin slice (using the rotating wheel saw IsoMet 4000 Buehler, Germany).

 For initial orthopaedic-preclinical evaluation of the biomimetic MSC-Scaffold for noncemented stemless RHA endoprostheses we have implanted 4 preprototypes of the MSC-Scaffold under the articular cartilage surface of medial and lateral femoral condyles of two laboratory swine, after the opening of their knee joints. One of the laboratory swine was a 9-month-old boar, weight of 85,5 kg, and the second was a 10-month-old boar, weight of 91,0 kg. Each animal received both implant variants of MSC-Scaffold preprototypes: varying with the distance between the spikes bases (100 *μ*m and 200 *μ*m, both circumferentially and radially) and with the external diameter of the MSC-Scaffold preprototype base (*ϕ*10 mm and *ϕ*15 mm, respectively). The surgery was carried in a veterinary clinic operating room with the permission of the Animal Ethics Committee in Poznan, Poland. During the surgical implantation of MSC-Scaffold preprototypes the general anesthesia by inhalation with endotracheal intubation and anesthetic monitoring were applied (given intravenously Cepetor 0,01–0,04 mg/kg BW i.v.; anesthesia was maintained with inhaler Isoflurane (Forane) controlled by pulse oximetry with heart monitor; Dräger AT-1): premedication, once given intramuscularly (in the same syringe) Cepetor/Medetomidine hydrochloride/(0,02–0,04 mg/kg BW i.m.) and Lewometadon (0,25–0,5 mg/kg BW i.m). Anteromedial slim incision of ca. 20 cm length over the operated right knee joint was done. Approach to the knee joint between the lateral margin of patella and the external side of patellar ligament and then between vastus lateralis muscle and rectus femoris muscle was applied. The articular capsule was opened on the lateral side of patella, and then the patella was dislocated medially. Hemostasis was done. Patellofemoral region of the knee joint was exposed. The implantation sites in both femoral condyles were prepared using a surgical drill. Subchondral holes were gradually widened until the final size with milling cutter to harbour the implant. During drilling and milling processes the bone holes were continuously cooled with saline. The holes in condyles were irrigated with saline, and bone debris was removed. The first preprototype of MSC-Scaffold was inserted into the medial femoral condyle, and the second preprototype was inserted into lateral femoral condyle. Implant insertion into the bone holes was performed using surgical impact. In Figure 7(a) two variants of MSC-Scaffold preprototypes (I, II) implanted under the articular cartilage surface of both femoral condyles are showed. Reposition of the patella on anatomical site was made. A layered suture of the wound was applied. Antibiotic regime after implantation was introduced: penicillin powder was given into the subcutaneous layer at the end of surgery, the wound was covered by a mesh impregnated with penicillin, and antiseptic dressing was applied; then after the surgery, Amikacin (Biodacyna) 1 g twice a day i.v. (or i.m.) for 3 days was given. On the third day after the surgery the swine were allowed for full weight bearing. Implants were kept for 6 weeks in the first operated animal and for 9 weeks in the second animal. Four-week postoperative radiological examinations were performed in the veterinary clinic with premedication of animals as by implantation, using the X-ray Stenoscop Plus, Mobile C-Arm (GE Medical Systems, Japan).

### 2.3. Harvesting of Bone Containing Implants

In 6 weeks and 9 weeks after implantation the explantation was performed in the veterinary clinic operating room (premedication and general anesthesia as by implantation), and the two knee joints with the MSC-Scaffold preprototypes were harvested from animals; the procedure was finished by euthanasia of the animals (Morbitan/*Pentobarbital natrium*/in lethal doses 200 mg/kg BW i.v.), according to the protocol approved by Animal Ethics Committee.

The bone-implant fragments were excised from distal femoral epiphysis and processed for histological analysis. From each bone-implant fragment the 1,5 mm thin slices were cut using the rotating wheel saw (IsoMet 4000 Linear Precision Saw, Buehler, Germany) under constant water irrigation (Figure 7(b)). Slices were cut along the direction parallel to the axis of central spike of the MSC-Scaffold preprototype. After that, the slices were fixed in 6 wt% formalin solution for 2 days. In the next step the slices of bone containing implant were decalcified using 4 wt% nitric acid (HNO_3_) for 24 hours. In the next step the MSC-Scaffold preprototypes were removed from bone slices. Then the bone slices were dehydrated in graded ethanol solutions (50 wt%, 60 wt%, 70 wt%, 95 wt%, and 100 wt% ethanol) and degreased in graded acetone solutions (90 wt% and 99,8 wt%). This was followed by a hydrophobic clearing agent (xylene) to remove the alcohol and finally infiltrated with molten paraffin wax to prepare bone specimens for cutting tissue slices on microtome. All the above processing stages starting from dehydration, each lasting 2 hours, were realized using the Semienclosed Benchtop Tissue Processor Leica TP1020 (Leica Microsystems GmbH, Germany). The peri-implant bone slices were embedded in paraffin wax and cut into 4 *μ*m thick sections. After performing reverse sequence of the above processes, the peri-implant bone sections were stained with haematoxylin-eosin and examined by light microscopy (Olympus CKX41, Olympus, Japan).

### 2.4. *In Vitro* Cytobiocompatibility (Biofunctionality) Tests

Normal human osteoblasts ((NHOst) Lonza, USA) were cultured on biomimetic MSC-Scaffold preprototypes for the evaluation of initial cell attachment and cell proliferation. Cells were cultured on MSC-Scaffold preprototypes in 12-well culture plates at initial seeding density of 5 × 10^4^ cells/well. Cells were plated in Dulbecco's Modified Eagle Medium with glucose and L-glutamine (PAA, Austria), 10% fetal bovine serum (PAA, Austria), 10 U/mL penicillin, and 10 U/mL streptomycin (Sigma, Germany) in 5% CO_2_ and 95% air atmosphere at 37°C (Galaxy 170R, New Brunswick, USA). The medium was changed every 48 hours and repeated until the cells reached a confluence state (10 days). Cells cultured in empty wells were used as control samples [31]. After 10 days of culture, the cell attachment and proliferation on biomimetic MSC-Scaffold preprototypes were analyzed by fluorescence microscopy (Olympus CKX41 with CKX-RFA fluorescence illuminator, Olympus, Germany); after staining in acridine orange (AO) solution, a typical 1–10 *μ*M AO staining concentration was used.

## 3. Results

**Figure 8:**
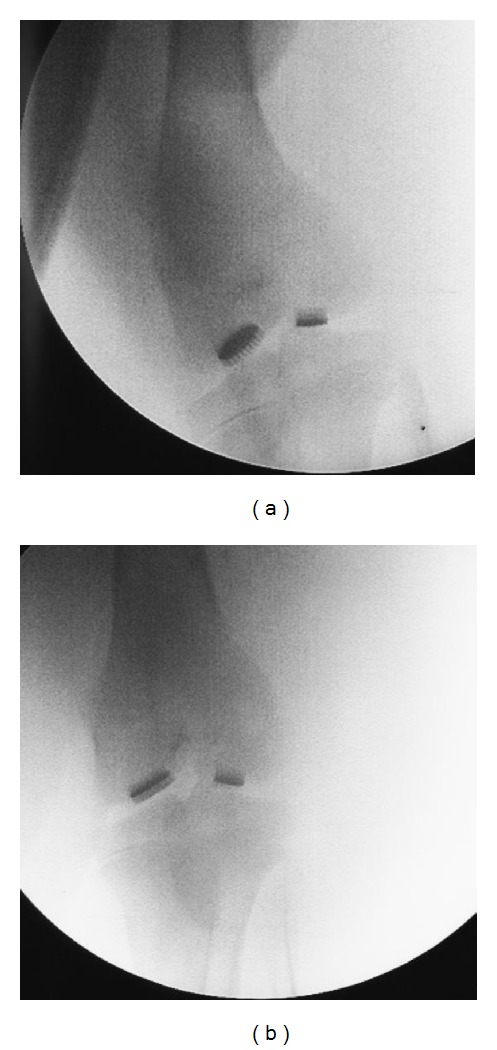
The anteroposterior radiograms at 4 weeks after implantation of two variants of preprototypes of MSC-Scaffold for noncemented stemless RHA endoprostheses implanted into femoral condyles of laboratory swine knee joints (using the X-ray Stenoscop Plus, Mobile C-Arm (GE Medical Systems), in veterinary clinic); radiographically and clinically no implant loosening signs nor migration after the surgery were observed.

The radiological examination of the MSC-Scaffold preprototypes implantation was performed at 4 weeks after surgery in the veterinary clinic. The anteroposterior radiograms of two variants of preprototypes of MSC-Scaffold for noncemented stemless RHA endoprostheses implanted into femoral condyles of laboratory swine knee joints are presented in Figure 8.

**Figure 9:**
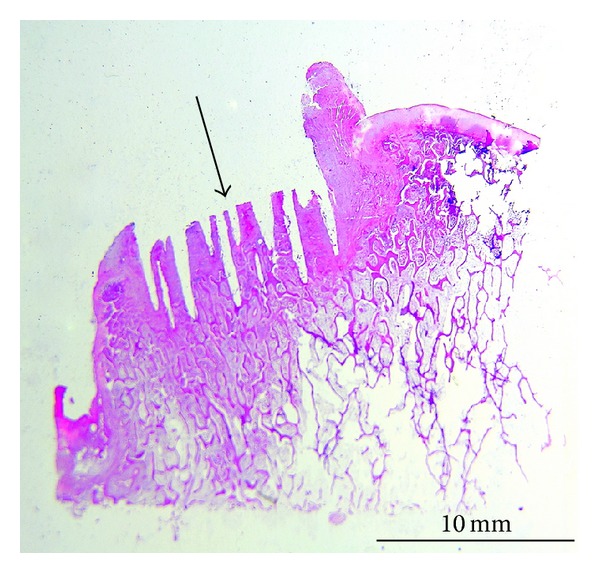
Exemplary histological section (H+E) of the peri-implant bone tissue after removing the MSC-Scaffold preprototype from bone-implant slices (see Figure 7(b)) after their decalcification showed smooth bone-implant contact surfaces (arrow), which suggests in sufficient osteointegration.

The exemplary histological section (H+E) of the peri-implant bone tissue—after removing the MSC-Scaffold preprototype from bone-implant slices (see Figure 7(b)) after their decalcification—is presented in Figure 9. It was found that the bone-implant contact surfaces were smooth, and the mechanical separation of implant from bone has not caused any pulling out of the peri-implant bone tissue.

**Figure 10:**
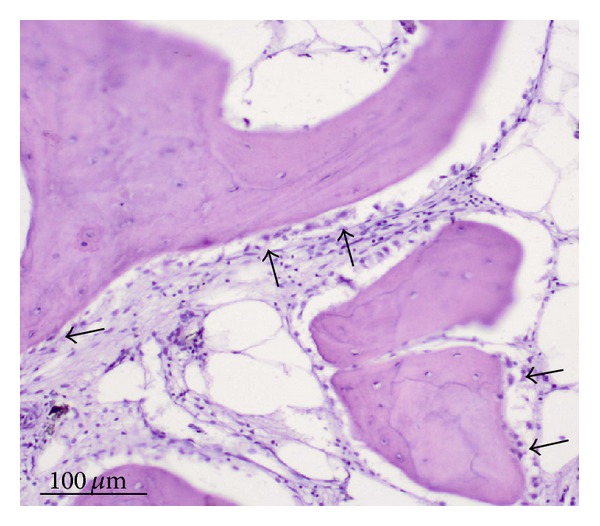
Relatively numerous osteoblasts (arrows) on bone trabeculae surfaces in periscaffold bone tissue histological sections (H+E staining) obtained from bone fragments containing implants harvested in the 6th week after the surgery.

No inflammatory morphological exponents (such as lack of neutrophils infiltration and lymphocytes, lack of macrophages aggregates) in the 6th week after the surgery histological sections of periscaffold bone tissue were found. Relatively numerous osteoblasts on bone trabeculae surfaces were noted in periscaffold bone tissue histological sections prepared from bone fragments containing implants harvested in the 6th week after the surgery, which means that the osteogenesis process is still running (Figure 10).

**Figure 11:**
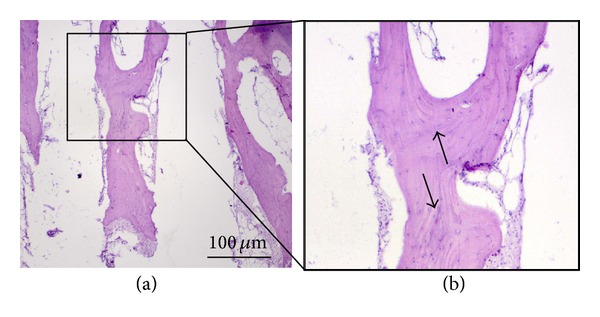
The 9th week after surgery histological sections showing the interspike pore space of the MSC-Scaffold preprototypes filled by matured trabecular bone tissue: bone trabeculae of periscaffold bone are considered as in equal age and mature as it is indicated by the clearly seen interlamellar lines and osteocytes in bone trabeculae (b).

Almost whole interspikes space of MSC-Scaffold on all the 9th week after surgery histological sections are filled by matured bone tissue (Figure 11(a)); no morphological exponents of osteogenesis process were found. Also no necrotic bone fragments which are produced during the first step of surgical implantation (see Section 1) were found in this space. Bone trabeculae of periscaffold bone in these histological sections are considered as in equal age and mature—as it is indicated by the clearly seen interlamellar lines and osteocytes in bone trabeculae (Figure 11(b)). 

**Figure 12:**
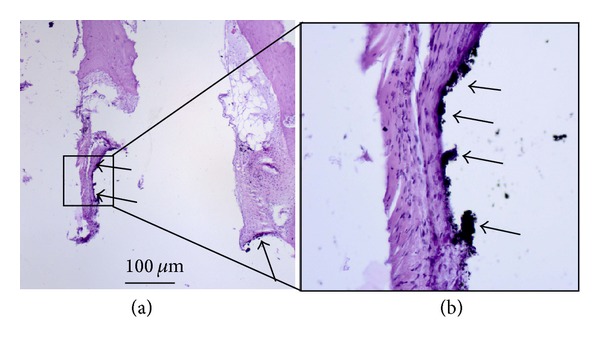
The 9th week after surgery histological sections (H+E) showing numerous metallic particles (arrows) being the remains after glass pearl blasting of spikes surfaces of MSC-Scaffold preprototypes manufactured in SLM technology.

**Figure 13:**
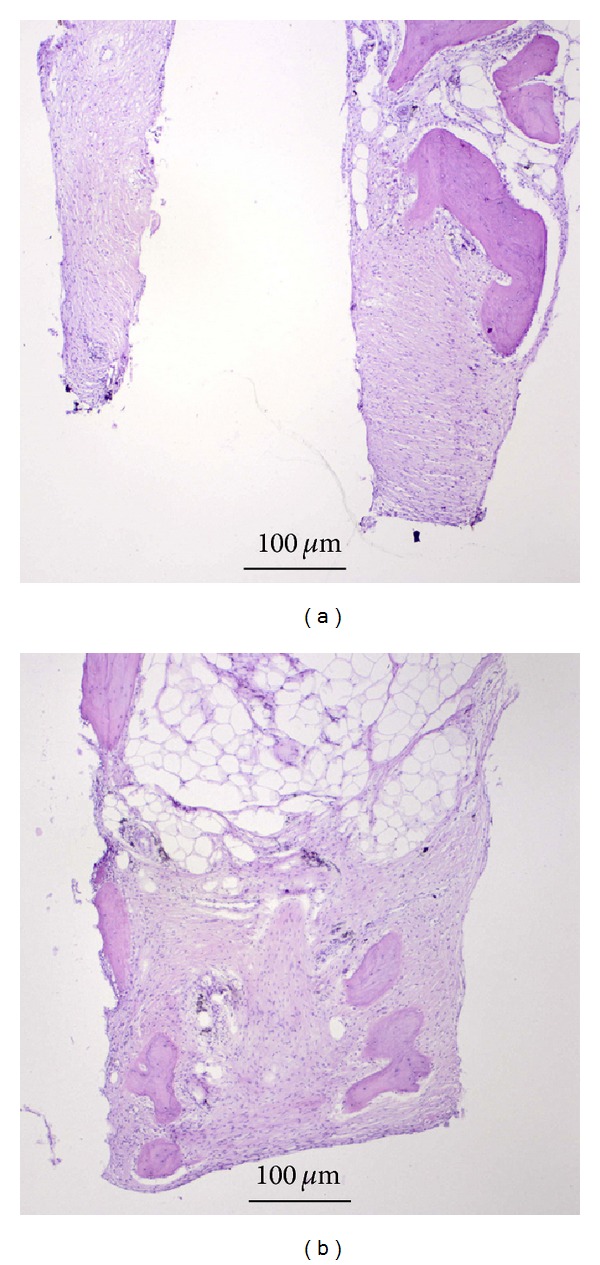
The 9th week after surgery histological sections (H+E) showing regions of the interspike space of MSC-Scaffold preprototype near the edges of the spikes bases (distance between spikes bases: (b) 100 *μ*m and (c) 200 *μ*m) filled almost completely by the fibrous connective tissue.

In the 6th and 9th weeks after surgery histological sections of periscaffold bone tissue we have noticed, especially in the interspike space of the scaffold near the edges of spike bases, the occurred numerous metallic particles (Figure 12(a), arrows, and Figure 12(b), higher magnification of the marked region) being the remains after glass pearl blasting of spikes surfaces of MSC-Scaffold preprototypes manufactured in SLM technology (cf.  Figure 6(d)). In the 9th week after surgery histological sections given in Figures 13(a) and 13(b), an exemplary region of the interspike space of MSC-Scaffold preprototype near the edges of the spikes bases, filled almost completely by the fibrous connective tissue, is shown.

**Figure 14:**
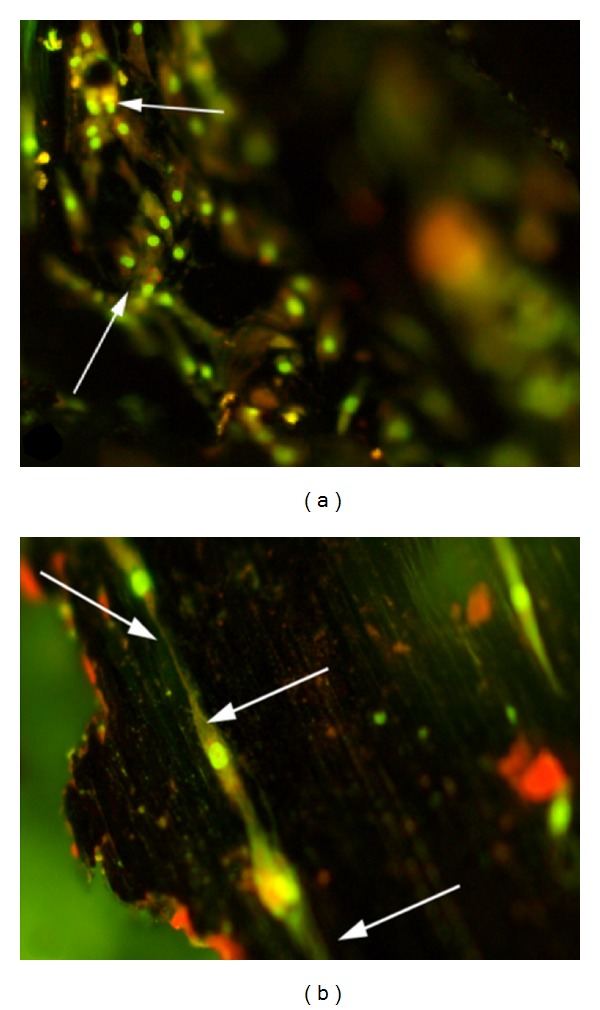
(a, b) Fluorescence microscopy after acridine orange (AO) staining of human osteoblasts ((NHOst) Lonza) after cultivation of the biomimetic MSC-Scaffold preprototypes for 10 days. Cells adhered to MSC-Scaffold's spikes and filled the space between them; they spread on the surface of the biomimetic MSC-Scaffold and start to contact each other via cytoplasmic extensions (white arrows) creating a three-dimensional cell-to-cell network. Thus, the preprototype spikes (mimicking the interdigitations of periarticular subchondral bone) can indeed act like a scaffold for osteoblasts.

The fluorescence microscopy photographs of the biomimetic MSC-Scaffold preprototypes after cultivation of human osteoblasts ((NHOst) Lonza) for 10 days are presented in Figure 14.

## 4. Discussion

The radiograms presented in Figure 8 showed that all four preprototypes were well situated in both femoral condyles in operated knee joints. No clinical sings of eventual loosening of implants were observed in both animals. In conclusion, no implant loosening, migration, or other early complications after the surgery were observed.

The smooth bone-implant contact surfaces showed at the exemplary histological section (Figure 9) indicate that the osseointegration of the spikes surfaces of the MSC-Scaffold preprototypes was not sufficient. The bone protein adsorption to implant surface occurring during the proper osseointegration would cause the pulling out of the peri-implant bone tissue together with removed implant, which was not observed. It is known that the bone protein adsorption to implant surface is better when the implant surface is modified with a biomimetic Ca-P (hydroxyapatite) coating [32]; our early results of modification of preprototypes of MSC-Scaffold surfaces with hydroxyapatite by cathodic electrodeposition process are presented in [33].

Following the 9th week after surgery histological sections observation (Figure 11) we can say that the scaffolding effect was obtained with our MSC-Scaffold preprototype, because its interspike pore space was filled by new formed and properly remodelled bone tissue providing primary biological fixation of the preprototype.

The absence of the bone tissue in the interspike space of the MSC-Scaffold preprototypes near the spikes base edges regions indicates that, due to the small distance between spikes base edges (100 *μ*m, Figure 12(b), and 200 *μ*m, Figure 12(c)), these regions are not sufficiently large to allow the bone tissue formation. In addition, the bone tissue formation in this region may be inhibited by the inflammatory process associated with the foreign body biological response on the remained metallic particles. This is the second indication followed from the performed experimental surgery to consider enlarging the distances between the spikes base edges in our next preprototypes of MSC-Scaffold for non-cementless stemless RA endoprostheses. Moreover, the enlarging of the interspike space near the spikes base edges will increase the effectiveness of the glass pearl blasting of spikes surface of the MSC-Scaffold preprototypes, and cleaning this region from the metallic particles remained after SLM manufacturing process.

It was found, as shown by the fluorescence microscopy (Figure 14(a)), that NHOst cells were attached to biomimetic MSC-Scaffold's spikes and filled the space between them. The cells have proper morphology and in contact with the material surface firstly attach, adhere, and then spread. The cells spreading on the surface of the biomimetic MSC-Scaffold start to contact each other via the cytoplasmic extensions, creating a three-dimensional cell-to-cell network (Figure 14(b), arrows). This result shows that the neighboring spikes of the biomimetic MSC-Scaffold preprototypes for RA endoprosthesis can indeed act like a scaffold (exhibit scaffolding effect) for human osteoblasts, and, thus, the biomimetic MSC-Scaffold preprototype potentially ensure the expected bone tissue ingrowth into its interspiked space *in vivo* with the following permanent fixation of RA endoprosthesis components in the surrounding bone tissue. The cells grow relatively more effectively between some spikes; however, the multispiked MSC-Scaffold material surface does not promote the intensive cell growth, which means that the surface of the biomimetic MSC-Scaffold contacting with bone should be physicochemically modified.

## 5. Final Remarks and Conclusions

So far, the worldwide accepted standard fixation method for RHA endoprostheses is a hybrid technique consisting in fixing the short-stem femoral component with cement in combination with an uncemented acetabular component. The extensive cement penetration into periarticular cancellous bone of femoral head always causes the regional blood supply insufficiency in this region, leading to the progressive weakening of the internal bone microstructure and often following the loosening of the femoral component of RHA endoprosthesis. Moreover, the stress shielding around the peri-implant short-stem femoral component often causes femoral neck fractures, which is the major reason (43% [22]) for revision of primary RHA endoprostheses, being in 62% of all cases of the femoral-only revision [22].

In this paper, the new fixation method for RHA endoprostheses by means of the biomimetic multispiked connecting scaffold (MSC-Scaffold) mimicking the interdigitations of periarticular subchondral bone is presented. Such connecting scaffold can generate the new type of RHA endoprostheses, that is, the stemless and fixed entirely without cement RHA endoprostheses. It is an alternative for the current short-stem and partly cemented RHA endoprostheses. The new fixation method invented by Rogala [23–25] and the MSC-Scaffold preprototype designed in our research team [27, 28] assumes more physiological load transmission in peri-implant bone in comparison to the traditional short-stem cemented RHA endoprostheses. The preprototypes of this MSC-Scaffold were manufactured with modern additive laser additive technology—the selective laser melting [29]. 

The initial pilot surgical implantations in animal model (two laboratory swine) of the MSC-Scaffold preprototypes have postoperatively (after two months) showed neither implant loosening, migration, and nor other early complications. The histopathological evaluation of the periscaffold spikes bone tissue has let us conclude that (1) the scaffolding effect was obtained with our MSC-Scaffold preprototype (because the majority of its interspike pore space was filled by new formed and properly remodeled bone tissue, providing primary biological fixation of the MSC-Scaffold preprototypes in periarticular cancellous bone) and (2) to improve the osseointegration the MSC-Scaffold material surface should be physicochemically modified, for example, with hydroxyapatite. These conclusions dealing with the bone scaffolding effect of our MSC-Scaffold preprototypes were also supported by the results obtained after 10-day culture of human osteoblasts on these preprototypes.

Some histopathological findings in the periscaffold domain (fibrous connective tissue and metallic particles near the MSC-Scaffold spikes base edges) prompt considering the necessity to optimize the design the MSC-Scaffold in the regions of its interspike space near the spikes base edges, to provide more room for new bone formation in this region. This is expected to increase the MSC-Scaffold preprototypes biointegration with periscaffold bone tissue by taking a full advantage of the interspike space of the MSC-Scaffold, which in turn will be translated into the improvement of the mechanical load transmission in bone-implant interface without micromotions. In addition, the conditions for the indispensable postprocessing (by glass pearl blasting) after the SLM manufacturing process of the MSC-Scaffold will be improved which will be translated into eliminating the undesired metallic particles adhered to the MSC-Scaffold spikes surfaces near the spikes base edges.

Other directives for optimization of our MSC-Scaffold preprototype design are expected to be concluded from results of the biomechanical tests during push-in of the MSC-Scaffold preprototypes into peri-articular cancellous bone and the numerical experiment of load transmission in the periscaffold bone, both carried simultaneously in the frames of our research project no. NN518412638.
